# Detecting the Intention to Move Upper Limbs from Electroencephalographic Brain Signals

**DOI:** 10.1155/2016/3195373

**Published:** 2016-04-27

**Authors:** Berenice Gudiño-Mendoza, Gildardo Sanchez-Ante, Javier M. Antelis

**Affiliations:** Tecnologico de Monterrey, Campus Guadalajara, Avenida General Ramón Corona 2514, 45201 Zapopan, JAL, Mexico

## Abstract

Early decoding of motor states directly from the brain activity is essential to develop brain-machine interfaces (BMI) for natural motor control of neuroprosthetic devices. Hence, this study aimed to investigate the detection of movement information before the actual movement occurs. This information piece could be useful to provide early control signals to drive BMI-based rehabilitation and motor assisted devices, thus providing a natural and active rehabilitation therapy. In this work, electroencephalographic (EEG) brain signals from six healthy right-handed participants were recorded during self-initiated reaching movements of the upper limbs. The analysis of these EEG traces showed that significant event-related desynchronization is present before and during the execution of the movements, predominantly in the motor-related *α* and *β* frequency bands and in electrodes placed above the motor cortex. This oscillatory brain activity was used to continuously detect the intention to move the limbs, that is, to identify the motor phase prior to the actual execution of the reaching movement. The results showed, first, significant classification between relax and movement intention and, second, significant detection of movement intention prior to the onset of the executed movement. On the basis of these results, detection of movement intention could be used in BMI settings to reduce the gap between mental motor processes and the actual movement performed by an assisted or rehabilitation robotic device.

## 1. Introduction

Brain-machine interface (BMI) systems are emerging technologies that provide a novel communication channel for both healthy people and patients with limited communication or motor impairments [1, 2]. A BMI system decodes the mental tasks performed by the user using brain signals recorded with invasive or noninvasive techniques. This is in turn used to control an application or an external device such as the computer cursor, a robotic wheelchair, or an orthosis device [3, 4]. As the peripheral nervous system is not involved in this process, a BMI can be a promising assistive technology for people with partial or complete motor disabilities [5, 6]. The most important application of BMI systems is the control of motor assisted robotic devices, which are employed for motor restoration or motor rehabilitation [7, 8]. This also includes rehabilitation scenarios based on virtual reality environments [9, 10]. These applications may provide patients who suffered stroke or spinal cord injury with the possibility of shortening the recovery period to improve their motor functioning.

The cue-based synchronous protocol is the traditional paradigm employed to decode mental tasks from the brain activity in BMI settings. In this paradigm, the mental task is first performed by the user and then the BMI technology applies decoding algorithms to identify the task [8]. Then, a control signal or command is provided to drive neuroprosthesis [11], for example, a real or virtual robotic arm. For instance, in a BMI based on motor imagery (MI), the user performs mental imagination of different limb movements and then the BMI technology identifies the moved limb which is used to provide a command of movement in the application [12]. Thus, the user's mental task is associated with the movement provided by the application. However, there exits an inherent delay between the time of the mental task and the time of the movement performed by the application. In consequence, the movements performed by external devices, either real or virtual, are not found natural by the user.

Reducing the temporal gap between mental motor tasks and the actual movement performed by an assisted or rehabilitation robotic device might be useful to obtain fast and natural motor control. This can also promote motor recovery at the cortical level [13, 14]. To achieve this early detection of movement information, this work studies the decoding of natural movement information before a motor task is performed. Several previous works have studied this kind of decoding from the noninvasive electroencephalographic (EEG) brain signals. These studies are based on movement related cortical potentials (MRCP), spectral power (SP), and common spatial patterns (SCP) features of the EEG to detect movement information preceding actual movements.  Table 1 presents a summary of the state of the art of the most relevant works devoted to the detection of movement intention. These studies have demonstrated the feasibility of detecting motor information before a movement is performed.

Nonetheless, more research is still required to achieve early detection of movement in more realistic situations. For this reason, this work proposed the continuous detection of movement intention in self-paced natural reaching movements of the upper limbs. This experimental task was chosen because it resembles the common daily-live-activity of grasping an object such as a glass of water or a bottle. It is important to detect the movement intention with sufficient preceding time in order to be able to use this information piece on time to provide natural movement control to users in BMI-based motor recovery and motor rehabilitation scenarios. Therefore, this study addresses the detection of the intention to move irrespective of the moved limb within a continuous decoding strategy. Six healthy right-handed subjects participated in the experimental sessions. The results revealed significant event-related desynchronization before and during the execution of the reaching movement task, and these cortical rhythms were used as features to continuously detect the intention to move the limbs. In addition, significant classification rate of movement intention was achieved before the onset of the executed reaching movements.

This paper is organized as follows: the description of the experiment, the data processing and analysis, and the evaluation process and metrics are detailed in Section 2; Section 3 describes the results, in particular the significant activity of event-related desynchronization/synchronization and the classification results oriented to detect movement intention; finally Section 4 presents the conclusions and future work.

## 2. Methods

### 2.1. Design and Execution of the Experiment

**Figure 1:**
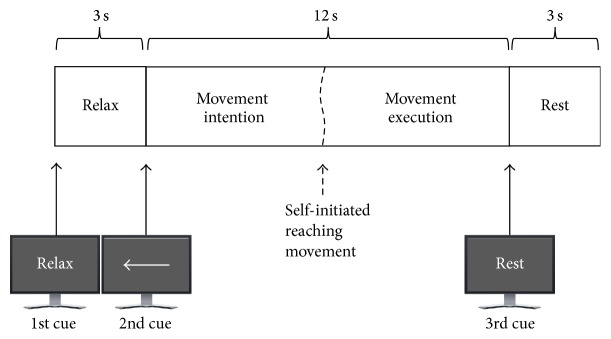
Temporal sequence of a trial with the three visual cues presented to the participants. Note that the second cue indicated self-initiating a reaching movement with the left arm.

The experiment consisted of self-paced natural reaching movements of the upper right/left limb. This experiment was selected because it resembles the common daily-live-activity of grasping an object such as a glass of water. Participants were comfortably seated with both forearms resting on the chair's arm and a computer screen was located in front of them. The experiment consisted of the execution of many repetitions or trials of reaching movements with either left or right arm and was guided by visual cues presented on the screen. Each trial consisted of three cues. The first cue showed the text “relax” for three seconds and indicated staying comfortably seated with the arms placed on the chair's arms in complete relaxation. Participants were requested not to execute or imagine any movement. The second cue showed for twelve seconds an image with an “arrow” pointing to the left/right and indicated moving naturally the corresponding arm towards the center of the screen. Participants were instructed not to initiate the movement immediately after the arrow was presented but to initiate it whenever they wish, waiting for at least five seconds while avoiding any mental count. Accordingly, the movement initiation varies across trials. Immediately after the reaching movement was completed, participants were instructed to return back the arm towards the chair's arms. The third cue showed the text “rest” and indicated resting, moving, or blinking for three seconds. Therefore, each trial lasted for eighteen seconds in total.  Figure 1 displays the full temporal sequence of a trial during the experiment. Participants were asked to avoid any movement and to minimize blinking from the presentation of the first cue and up to the termination of the reaching movement.

The experiment was executed in four blocks of 24 trials (7.2 min per block) resulting in a total of 96 trials (28.8 min for all blocks). To avoid fatigue, patients could rest between blocks as long as they needed. To keep balance of the number of trials for the left and right arm, each block contained the same number of left and right movements, which were presented in a pseudorandom manner. This experiment was approved by the ethics committee of the university.

### 2.2. Participants

Six able-bodied right-handed subjects (two males and four females; age range 23–19 years; mean ± std 20.33 ± 1.51 years) without diagnosis of neurological or motor disease voluntarily participated in this study. All participants were students from the university and did not have experience with electroencephalogram (EEG) recording protocols or brain-machine interface (BMI) experiments. They were duly informed about the objective of the research and the experimental procedure and all of them signed informed consent forms. They were informed that they could leave the experiment when they wanted.

### 2.3. Recording of EEG and EMG Signals

EEG signals were recorded using monopolar electrodes at 21 scalp positions according to the 10/10 international electrode location system. EEG signals were recorded from scalp locations *Fp*1, *Fp*2, *F*7, *F*3, *Fz*, *F*4, *F*8, *T*3, *C*3, *Cz*, *C*4, *T*4, *T*5, *P*3, *Pz*, *P*4, *T*6, *O*1, *O*2, *A*1, and *A*2, with the ground at *Fpz* and the reference at the left earlobe. EMG signals were recorded with bipolar electrodes located above the biceps brachii muscle and the triceps brachii muscle. These EMG signals were recorded from both arms and they were used to establish the time of the movement initiation of each trial. EEG and EMG data were recorded at a sampling frequency of 2048 Hz and no filtering was applied. The electrode impedance was kept below 5 k*Ω* for EEG and 20 k*Ω* for EMG. EEG and EMG signals were simultaneously recorded using a Nexus-32 electrophysiology monitoring system from Mind Media. BioTrace+ software was used to manage the presentation of the visual cues and the recording of the EEG and EMG signals and to store the data for offline processing.

### 2.4. Data Preprocessing

After the experimental sessions, recorded data were subjected to offline preprocessing and analysis. EEG and EMG data were trimmed from the presentation of the first cue up to the presentation of the third cue; thus, the resulting trials lasted for fifteen seconds. Then, the time latency of the movement onset of each trial was computed with the EMG activity following this procedure: (i) the EMG signal from the moved arm was selected; (ii) this EMG signal was high-pass filtered with a cutoff frequency of 10 Hz using a sixth-order Butterworth-type infinite impulse response (IIR) filter; (iii) the Hilbert transform was computed from the filtered signal; (iv) the magnitude of the Hilbert transform was smoothed and normalized *z*-score; (v) the first value greater than zero in the resulting signal was defined as the EMG-based movement onset. Trials for which the movement onset was lower than 3 s (early arm movement initiation) and greater than 11 s (delayed arm movement initiation) relative to the presentation of the second visual cue were discharged. Then, the time axis of each trial was rereferenced to the EMG-based movement onset; that is, *t* = 0 represents the initiation of the reaching movement. Finally, trials were trimmed from the initiation time *t*_ini_ (i.e., *t*_ini_ is the time of the presentation of the first cue) up to 1 s relative to the EMG-based movement onset.

Frontal electrodes (*Fp*1 and *Fp*2), electrodes located near to the neap (*O*1 and *O*2), and other electrodes far away from the motor cortex (*F*7, *F*8, *T*3, *T*4, *T*5, *T*6, *A*1, and *A*2) were removed from all participants as they are usually contaminated by eye blinks, muscle activity, and other artifacts; thus nine electrodes located on or surrounding the motor cortex (*F*3, *Fz*, *F*4, *C*3, *Cz*, *C*4, *P*3, *Pz*, and *P*4) were kept and used for the subsequent analysis. EEG data was resampled to 256 Hz, filtered from 0.1 Hz to 100 Hz using a zero-phase, four-order, bandpass Butterworth filter, and rereferenced using the common average reference (CAR) filter where the average across all channels is subtracted for each channel independently for each time sample.

### 2.5. Event-Related Desynchronization/Synchronization

To compute the significant event-related desynchronization/synchronization of each electrode, a bootstrap analysis of the time-frequency representation was performed. The goal in this analysis was to study the underlying task-related oscillatory brain activity during intention of motion [15]. All trials were trimmed from −6 to 1 s relative to the EMG-based movement onset. This allows all trials to have the same length. For each trial and every channel, the time-frequency representation TFR(*t*, *f*) was computed in the frequency band [2, 40] Hz at the resolution of 1 Hz using Morlet wavelets [16]. For each channel individually, the event-related desynchronization/synchronization (i.e., power increase/decrease) relative to the baseline [−6, −3) s was computed for each time and frequency as ERDS(*t*, *f*) = 100 × (TFR(*t*, *f*) − TFR_baseline_(*f*))/TFR_baseline_(*f*), where TFR_baseline_(*f*) is the average of TFR(*t*, *f*) in the baseline interval for frequency *f*. The significant event-related desynchronization/synchronization of each channel was computed with a bootstrap analysis following [17] at the significant level of *α* = 0.05.

### 2.6. Detection of Movement Intention

Detection of movement intention was based on spectral power features and on a support vector machine (SVM) used to distinguish between* relax* and* intention*.

#### 2.6.1. Features

Spectral power features were computed with an autoregressive spectrum (ARS) model of order 16 [18, 19], where Burg's method was employed to estimate the model coefficients and the noise variance [20]. For each electrode, only values of the spectral power in the motor-related *α*[8, 14] Hz and *β*[14, 25] Hz frequency bands were used. Spectral power values were computed at the resolution of 1 Hz. This resulted in 18 spectral power values per electrode. Thus, the feature vector is** x **∈*ℝ* ^*D*^ where *D* = 162 (18 spectral power values ×  9 electrodes), which is associated with a class label *y* ∈ {relax, intention}. For a given time instant *t* where *t* ∈ [*t*_ini_, 1], the spectral power features are computed from the EEG in the time window [*t* − *T*, *t*], where *T* is the size of the window. Note that the time *t* corresponds to the endpoint of the used time window; therefore the computed features were causal.

#### 2.6.2. Classifier

**Figure 2:**
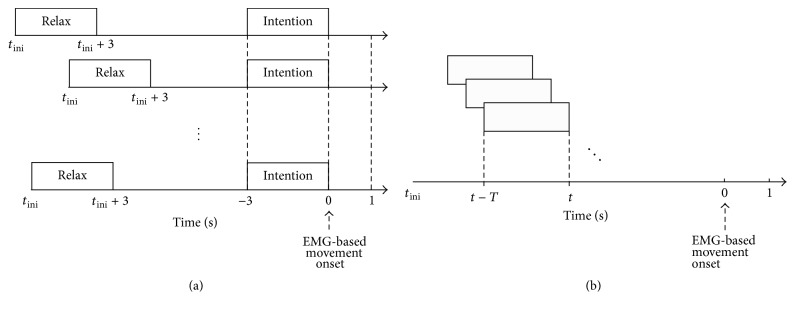
Illustration of the data segments used to train and to test the classification. (a) Data segments in the relax phase and the movement intention phase used to extract features to train the classifier. (b) Data segments used to carry out classification in a test trial.

To discriminate between relax and intention, a SVM with a radial basis function (RBF) kernel [21] was employed. The implementation of the SVM relied on the LIBSVM library [22]. The hyperparameters of the RBF were *C* = 1 for the regularization parameter and *σ* = 0.5 for the width [23, 24]. To train this classifier, the features were extracted exclusively from the relax phase [*t*_ini_, *t*_ini_ + 3] and the movement intention phase [−3, 0] and they were labeled as relax and intention, respectively.  Figure 2(a) illustrates the segments of relax and intention used to extract features. Within these segments, the features were computed from nonoverlapping time windows of length *T*. In the relax phase features were computed at *t*_*k*_ = *t*_ini_ + 3 − *kT* for *k* = 0,1, 2,… provided that *t* ∈ [*t*_ini_, *t*_ini_ + 3]. In the movement intention phase features were computed at *t*_*k*_ = −*kT* for *k* = 0,1, 2,… provided that *t* ∈ [−3, 0]. Prior to training, features were *z*-score normalized according to *x*_*i*_ = (*x*_*i*_ − *μ*_*i*_)/*σ*_*i*_, (*i* = 1,2,…, *D*), where *μ*_*i*_ and *σ*_*i*_ are the corresponding mean and standard deviation of the *i*th feature computed exclusively from training data.

#### 2.6.3. Evaluation Procedure and Metrics

Detection of movement intention was assessed for each subject independently following this procedure:

(i) Randomly select 80% of the trials as training set and use remaining 20% as test set.
(ii) For each trial of the training set, extract the spectral power based features from the relax phase and the movement intention phase according to *T* and then train the classifier.
(iii) Apply the classifier to each trial in the test set using sliding windows of size *T* in steps of 0.1 s (Figure 2(b) illustrates the process employed to perform classification in a test trial).
(iv) Compute performance metrics using the entire test set.

The following metrics were considered: (i) classification accuracy (CA) (rate of correct classifications achieved within the relax [*t*_ini_, *t*_ini_ + 3] and the intention [−3, 0] s phases), true positive events (TPE) (movement intention detection rate obtained in the intention phase), and true negative events (TNE) (relax detection rate obtained in the relax phase); (ii) time-resolved movement intention detection accuracy or DA(*t*) (rate of movement intention detected at time *t*); (iii) time instant of the movement intention onset or tMI (the lowest time instant for *t* < 0 at which significant differences between DA(*t*) and the chance level are unequivocally achieved); and (iv) trials where movement intention is detected or NT_D_ (rate of trials where movement intention was unequivocally detected prior to movement initiation, i.e., *t* < 0).

This evaluation procedure was repeated 30 times and distributions and the mean ± std of the performance metrics were computed for each participant and for all of them. The significant chance level of the detection accuracy or DA_sig_(*t*) was computed empirically by randomly permuting the class labels during the training of the classifier. This procedure was conducted also 30 times for each subject using 80% of the trials for training (with random labels) and remaining 20% for evaluation. The significant chance level of the classification accuracy or CA_sig_ was computed as the maximum empirical chance level in the relax [*t*_ini_, *t*_ini_ + 3] and intention [−3, 0] s phases. To examine significant differences between the distributions of CA and the significant chance level CA_sig_ the Wilcoxon signed rank test was used, while to examine significant differences between DA(*t*) and DA_sig_(*t*) the Wilcoxon rank-sum test was employed. These statistical tests were performed at a confidence level of *α* = 0.01.

## 3. Results

The time instant of the movement initiation computed with the EMG activity was estimated in all the trials of all subjects after the presentation of the second visual cue (i.e., the one instructed to self-initiate the reaching movement of the left/right arm). Movement onset was lower than 3 s in 3% of the trials while it was greater than 11 s in 1% of the trials. These trials were discharged and not used in the rest of the work. Then, the total number of trials across all subjects used in this study was on average 92.83 ± 2.79 (minimum of 89 and maximum of 96).  Table 2 shows a summary of the estimated EMG-based movement onset for all subjects and the average for all of them. The average movement onset across all subjects was 7.04 ± 1.42 s (minimum of 3.18 s and a maximum 10.82 s).

**Figure 3:**
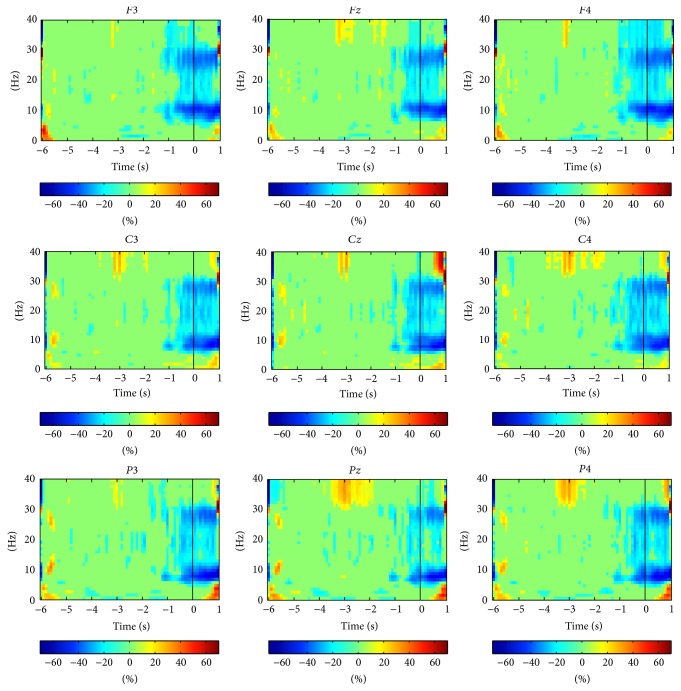
Significant event-related desynchronization/synchronization activity computed for all trials and subjects. Horizontal axis represents time (units of s) while vertical axis represents frequency (units of Hz). Solid black lines in all graphs represent *t* = 0 s or the movement onset. Significant desynchronization (*p* < 0.05) is observed in all sensors in the motor-related *α*[8,13] Hz and *β*[14,30] Hz frequency bands from *t* ≈ −1.5 s, while no significant desynchronization or synchronization (*p* > 0.05) is observed before *t* ≈ −1.5 s.

The significant activity of event-related desynchronization/synchronization computed across all trials and subjects is presented in Figure 3. Significant desynchronization (*p* < 0.05) is observed in all sensors and in the motor-related *α*[8, 13] Hz and *β*[14, 30] Hz frequency bands around the movement onset *t* = 0 s. This significant desynchronization starts in the movement intention phase roughly at 1 s prior to the movement onset and remains significant up to the movement execution interval *t* ≥ 0. No significant desynchronization or synchronization (*p* > 0.05) is observed before ≈−1 s. Note that the significant desynchronization is uniformly distributed in all sensors and in both hemispheres; that is, no spatial pattern of desynchronization/synchronization is observed across the motor cortex.

The average of significant event-related desynchronization/synchronization in the *α*[8, 13] Hz and *β*[14, 30] Hz frequency bands of each electrode was computed over time windows along the entire duration of the trial. These results are presented in Table 3. In all electrodes, the significant desynchronization is absent for time windows from [−6, −5) to [−4, −3) s but then begins to intensify gradually from [−3, −2) s up to [0, 1) s. Thus, the significant desynchronization starts prior to the movement onset, that is, at the movement intention phase, and remains significant up to the movement execution phase. Note that the event-related desynchronization/synchronization in the *α*/*β* frequency bands averaged for all electrodes is −4.72/−3.84, −24.53/−16.21, and −39.90/−20.44 for time windows [−2, −1), [−1,0) and [0,1) s, respectively. This shows that the significant desynchronization is more prominent during the movement execution phase than during the movement intention phase and that it is stronger in *α*[8, 13] Hz than in the *β*[14, 30] Hz frequency band.

**Figure 4:**
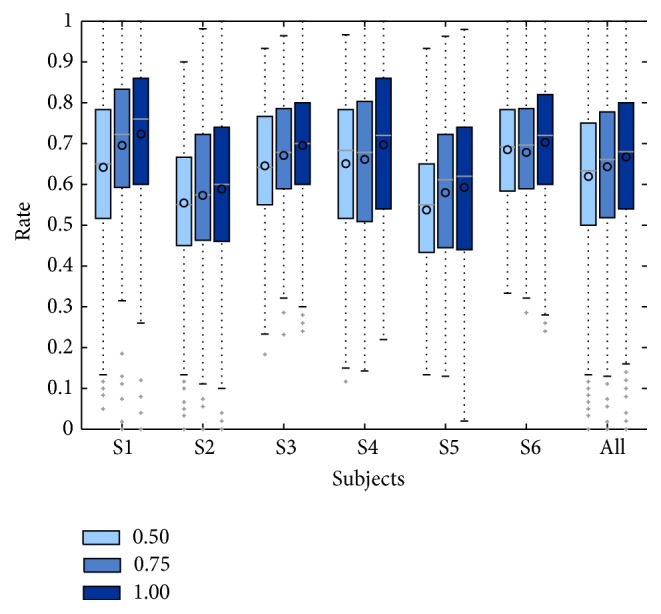
Distribution of the classification accuracy CA obtained for time windows *T* = 0.5, 0.75, and 1. Results are clustered by subject and the last group of boxplots present the CA for all subjects. Horizontal line over each boxplot represents the median, meanwhile the circle represents the mean.

The first classification analysis explored the impact of the window size *T* used to compute the spectral power features to classify between relax and intention.  Figure 4 shows, for each subject and for all of them, the distributions of the classification accuracy metric CA for window sizes of  *T* = 0.5, *T* = 0.75, and *T* = 1 s and the significant chance level CA_sig_ (maximum chance level across all subjects achieved in the relax and movement intention phases). For subjects 1, 3, 4, and 6, the median of the distribution for all *T* is greater and significantly different than the chance level CA_sig_ (*p* < 0.01, Wilcoxon signed rank test). However, for subjects 2 and 5, no significant differences were found between the median of the distributions and the chance level CA_sig_ (*p* > 0.01, Wilcoxon signed rank test). The averages of CA for *T* = 0.5 s were 0.64 ± 0.18, 0.55 ± 0.16, 0.65 ± 0.14, 0.65 ± 0.17, 0.54 ± 0.16, and 0.68 ± 0.14, for *T* = 0.75 s were 0.70 ± 0.18, 0.57 ± 0.19, 0.67 ± 0.14, 0.66 ± 0.18, 0.58 ± 0.18, and 0.68 ± 0.14, and for *T* = 1 s were 0.72 ± 0.19, 0.59 ± 0.20, 0.70 ± 0.16, 0.70 ± 0.19, 0.59 ± 0.20, and 0.70 ± 0.16, respectively, for subjects 1 to 6. The results across all subjects showed that the median of the distributions of CA for all *T* is also greater and significantly different than the chance level CA_sig_ (*p* < 0.01, Wilcoxon signed rank test).  Table 4 summarizes the results of classification accuracy (CA), true positive events (TPE), and true negative events (TNE) obtained across all subjects for the three windows sizes. The averaged values of CA for *T* of 0.5, 0.75, and 1 were 0.62 ± 0.06, 0.64 ± 0.05, and 0.67 ± 0.06, respectively, while TPE/TNE were 0.62/0.62, 0.65/0.64, and 0.67/0.66, respectively. These results show that the performance in the recognition between relax and intention increases as the time window size *T* increases. Therefore, a window size *T* = 1 s was used in the rest of this work to study the detection of movement intention.

**Figure 5:**
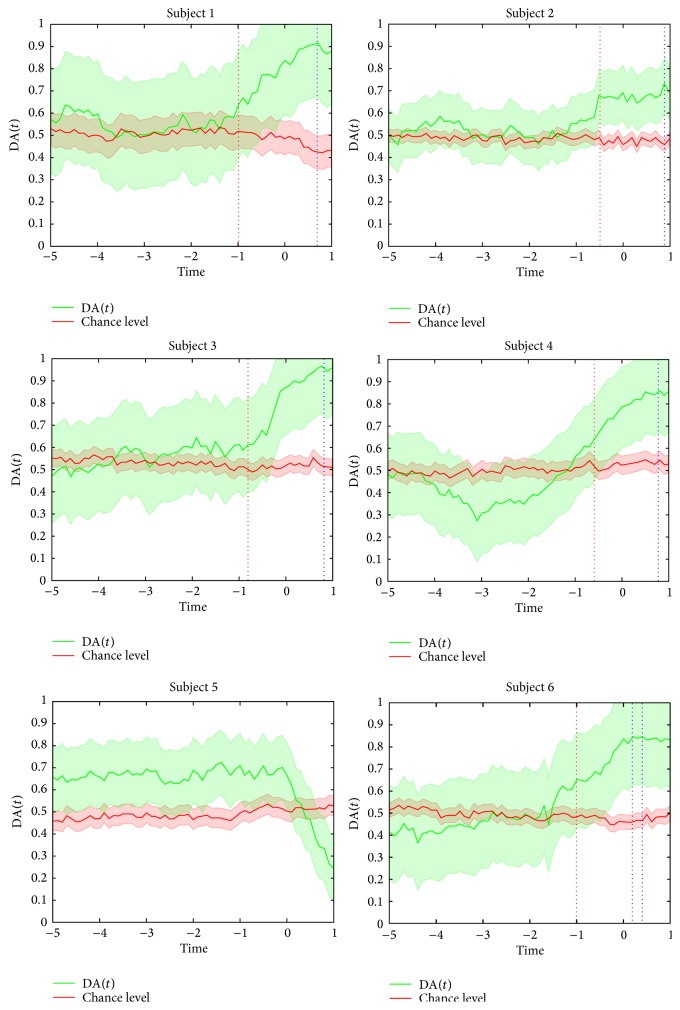
Time-resolved movement intention detection accuracy DA(*t*) (solid green line) and the empirical significant chance level of detection accuracy DA_sig_(*t*) (solid red line) of each subject. *t* = 0 refers to the initiation of the reaching movement. Shaded regions bounding the curves indicate the standard deviation. Vertical dotted blue lines represent the time of the maximum DA(*t*). Vertical dotted red lines represent the tMI.

Figure 5 shows the time-resolved detection accuracy DA(*t*) and the significant chance level of the detection accuracy DA_sig_(*t*). Results are presented for each subject separately. In all subjects DA(*t*) is presented from *t* = −5 s. This is due to the following: first, the trial's initiation time *t*_ini_ is different across all subjects and trials and the common initiation time across all of them is *t* = −6 s and, second, the window size used to compute the causal features is *T* = 1 s. For all subjects (except number 5), DA(*t*) is initially at the chance level and starts to rise before the movement initiation at around *t* = −1 s. In other words, no movement intention is detected from −6 to ≈−1 s while detection of movement intention is observed from ≈−1 s. The maximum DA(*t*) is 0.92, 0.73, 0.97, 0.86, and 0.85 for subjects 1 to 6, respectively, (excluding subject 5). These peaks of detection accuracy are achieved at *t* = 0.7, *t* = 0.9, *t* = 0.8, and *t* = 0.8, for subjects 1 to 4 and for subject 6 the maximum is reached in *t* = 0.2 and *t* = 0.4 (see vertical dotted blue lines in all plots of figures). Note that DA(*t*) always peaks at the movement execution phase *t* > 0. For subject 5, DA(*t*) is above chance level from −6 to ≈0 s and suddenly drops at about *t* = 0 s. This indicates that movement intention is always detected, even before the movement intention phase *t* < −3 s (i.e., it is not possible to discriminate between movement intention and no movement intention) and that movement intention is at the chance level at the movement execution phase *t* > 0 s. Thus, no movement intention information was detected for this participant. This result agrees with the distribution and average values of classification accuracy CA presented in Figure 4 for *T* = 1 where subject 5 presented the lower performance.

The fraction of trials where movement intention was detected prior to movement initiation NT_D_ and the time of movement intention detection tMI are summarized in Table 5. tMI may also be observed in Figure 5. These metrics were not computed for subject 5 as no significant time-resolved detection accuracy DA(*t*) was achieved for this participant. On average, movement intention was detected in 80 ± 0.7% of the trials across all subjects (minimum 69%, maximum 87%). These results reveal that movement intention is detected in the majority of the reaching movements performed by the participants. In addition, the time instant of movement intention detection is 0.78 ± 0.23 s prior to movement initiation (minimum −0.5 s, maximum −1.00 s).

## 4. Conclusions

This work proposed the continuous detection of movement intention from electroencephalographic (EEG) brain signals during natural self-paced reaching movements of the upper limbs. In the context of this work, movement intention was defined as the mental motor task (with no physical output) that occurs before the initiation of a movement, for example, motor planning. Six healthy subjects participated in this study and the EEG and electromyographic (EMG) activities were recorded.

The event-related synchronization/desynchronization of the EEG activity showed significant task-related cortical rhythms that started before the movement initiation, that is, at the movement intention phase, and remained during movement execution. Significant desynchronization (*p* < 0.05) was observed in the motor-related *α*[8, 13] Hz and *β*[14, 30] Hz frequency bands in all the selected sensors which were located above the motor cortex. This significant power decrease started about 1 s before the initiation of the movement and remains significant up to the movement execution, while no significant synchronization/desynchronization (*p* > 0.05) was observed before this time. This significant desynchronization was detected in both hemispheres and was consistent with the experimental motor task which includes reaching movements with either the left or right arm.

These task related cortical rhythms were then used to investigate the feasibility of discriminating between the relax phase and the movement intention phase. Therefore, biclass classification between relax and intention was evaluated using the spectral power of the ongoing EEG activity and a support vector machine as classifier. This classification was evaluated using different window sizes *T* of EEG to compute the spectral power features. The results showed that classification accuracy between the relax and the intention phases increases as the time window size *T* increases. Thus, a window size of *T* = 1 s was selected to compute the spectral power features used to study the continuous detection of movement intention.

Finally, significant time-resolved detection accuracy was obtained in 5 out of the 6 participants before actual movement initiation. In one of the participants, it was not possible to distinguish between movement intention from relax. The significant detection of movement intention starts to rise at about 1 s before the onset of the movement and remained during the movement execution phase. The time-resolved detection accuracy reached the maximum during movement execution. This agrees with the observed significant desynchronization activity reported above. The initial time instant of movement intention was on average 0.78 s, that is, almost half a second before the actual movement, which was detected in 80% of the trials. The proposed detector of movement intention could be used in BMI-based robot-assisted rehabilitation scenarios. The advantage would be the reduction of the temporal delay between mental motor processes and the actual movement performed by the robotic devices. This could in principle provide fast, natural, and continuous motor control that enhances and promotes motor relearning at the cortical level.

The next steps for this research are (i) detection of the moved arm, (ii) determination of novel features based on the estimation of spikes, (iii) testing a novel classifier based on lattice neural networks with dendritic processing, and (iv) performing feature reduction and selection using Fast Correlation Based Filter (FCBF) [25, 26] or sequential forward selection (SFS).

## Figures and Tables

**Table 1:** Description of the state of the art of works reporting decoding of motor information preceding actual movements from electroencephalographic brain signals. ME: motor execution; MI: motor imagery; MRCP: movement related cortical potentials; LDA: linear discriminant analysis.

	State-of-the-art movement intention detection from EEG						
	Participants	Motor task	EEG features	Classifier	Decoding	Accuracy	Detection time
[27]	8: healthy	ME/MI	Spectral power	Bayesian	Static	Varied between subjects	—

[28]	4: stroke	ME	Spectral power	LDA	Static	—	From −200 to −600 ms

[29]	6: healthy	ME	Spectral power	Bayesian	Static	62.9 ± 7.5%	—

[30]	19: healthy 5: stroke	ME/MI	MRCPs	Template matching	Continuous	Healthy: ME: 82.5 ± 7.8%, MI: 64.5 ± 5.33%; stroke: ME: 55.01 ± 12.01%	Healthy: ME: −66.6 ± 121 ms

[31]	20: healthy 5: stroke	ME/MI	MRCPs	Template matching	Continuous	Healthy: ME: 69 ± 21%, MI: 65 ± 22%; stroke: ME: 58 ± 11%	—

[32]	10: healthy 2: stroke	ME/MI	MRCPs	LDA	Continuous	—	−500 ms

[33]	10: healthy 2: stroke	ME	MRCPs	LDA	Continuous	Healthy: 76%; stroke: 47%	−312.5 ms

[34]	20: healthy	ME	Common spatial patterns	LDA	Static	83% in 12 subjects	—

[35]	6: healthy 3: SCI	ME	Spectral power and MRCPs	SDA	Continuous	From 75% to 40%	From −421 ms to −256 ms

**Table 2:** Summary of the estimated EEG-based movement onset for all subjects and the average across all of them. It only includes trials for which the movement onset was estimated from *t* ≥ 3 and *t* < 11 with respect to the presentation of the second visual cue which instructed to self-initiate the reaching movement of the left/right arm.

	EMG-based movement onset (s)			
	Mean	Std	Min	Max
S1	6.19	1.37	3.18	9.35
S2	7.54	1.30	4.04	10.36
S3	7.52	1.39	4.27	10.82
S4	6.69	1.30	3.55	10.00
S5	6.93	1.29	3.77	9.66
S6	7.38	1.30	4.07	10.41

Avg	7.04	1.42	3.19	10.82

**Table 3:** Across all subjects average of significant event-related desynchronization/synchronization in the motor-related *α*[8, 13] Hz and *β*[14, 30] Hz frequency bands over several time windows from −6 s to 1 s. Results are presented for each electrode and the last row presents the integrated values for all channels.

		Time window (s)						
		[−6, −5)	[−5, −4)	[−4, −3)	[−3, −2)	[−2, −1)	[−1, 0)	[0, 1)
*F*3	*α*	2.12	0.00	0.00	−0.59	−8.09	−28.66	−40.04
	*β*	0.79	−2.23	0.26	−1.13	−0.83	−15.89	−18.23

*Fz*	*α*	1.76	0.00	0.00	0.00	−3.99	−22.92	−37.74
	*β*	0.99	−1.05	0.20	−1.16	−1.34	−13.88	−16.39

*F*4	*α*	2.07	−0.22	0.00	0.00	−5.82	−28.64	−41.14
	*β*	1.62	−0.70	−1.07	−0.17	−3.58	−16.18	−17.45

*C*3	*α*	4.92	−0.39	0.00	0.00	−4.53	−23.90	−38.85
	*β*	0.31	−1.24	0.22	−1.50	−2.56	−14.74	−21.39

*Cz*	*α*	4.43	−0.38	0.00	−0.37	−4.47	−23.44	−40.67
	*β*	1.36	0.10	0.00	−2.17	−3.97	−15.19	−19.10

*C*4	*α*	3.36	−0.20	0.00	−0.77	−5.17	−26.23	−40.19
	*β*	0.60	0.42	0.00	−2.87	−5.01	−18.70	−20.82

*P*3	*α*	4.70	−0.20	0.00	−0.44	−6.86	−21.79	−42.01
	*β*	1.01	−1.06	−1.14	−3.64	−2.41	−16.42	−16.79

*Pz*	*α*	4.25	0.00	0.27	−0.21	−4.38	−16.06	−36.00
	*β*	0.57	−0.26	−2.00	−2.52	−1.71	−11.28	−9.26

*P*4	*α*	4.09	0.00	0.00	0.00	−5.02	−21.56	−41.10
	*β*	0.73	0.04	−0.80	−0.94	−1.48	−16.26	−16.55

Avg	*α*	4.24	−0.32	0.00	−0.38	−4.72	−24.53	−39.90
	*β*	0.76	−0.24	0.07	−2.18	−3.84	−16.21	−20.44

**Table 4:** Summary of the classification accuracy, true positive events, and true negative events achieved for all subjects for different window sizes *T* = 0.5, *T* = 0.75, and *T* = 1.0 s.

	Classification accuracy				
	Mean	Min	Max	TPE	TNE
0.50	0.62 ± 0.06	0.54	0.68	0.62	0.62
0.75	0.64 ± 0.05	0.57	0.70	0.65	0.64
1.00	0.67 ± 0.06	0.59	0.72	0.67	0.66

**Table 5:** Rate of trials where movement intention was unequivocally detected (NT_D_) and the time instant of the movement intention detection (tMI).

	Movement intention detection		
	NT_D_	1 − NT_D_	tMI
S1	0.84	0.16	−1.0
S2	0.69	0.31	−0.5
S3	0.87	0.13	−0.8
S4	0.79	0.21	−0.6
S5	—	—	—
S6	0.84	0.16	−1.0

Avg	0.80 ± 0.07	0.20	−0.78 ± 0.23
